# Diagnostic Performance of a Magnetic Resonance Imaging-directed Targeted plus Regional Biopsy Approach in Prostate Cancer Diagnosis: A Systematic Review and Meta-analysis

**DOI:** 10.1016/j.euros.2022.04.001

**Published:** 2022-05-02

**Authors:** Marinus J. Hagens, Mar Fernandez Salamanca, Anwar R. Padhani, Pim J. van Leeuwen, Henk G. van der Poel, Ivo G. Schoots

**Affiliations:** a Department of Urology, Amsterdam University Medical Centers VUmc, Amsterdam, The Netherlands; b Department of Urology, Netherlands Cancer Institute-Antoni van Leeuwenhoek Hospital, Amsterdam, The Netherlands; c Prostate Cancer Network Netherlands, Amsterdam, The Netherlands; d Department of Radiology, Netherlands Cancer Institute-Antoni van Leeuwenhoek Hospital, Amsterdam, The Netherlands; e Department of Radiation Oncology, Netherlands Cancer Institute-Antoni van Leeuwenhoek Hospital, Amsterdam, The Netherlands; f Department of Radiology and Nuclear Medicine, Erasmus University Medical Center, Rotterdam, The Netherlands; g Paul Strickland Scanner Centre, Mount Vernon Cancer Centre, Northwood, UK

**Keywords:** Prostate cancer, Prostate biopsy, Regional biopsies, Systematic biopsies, Diagnostic accuracy

## Abstract

**Context:**

Systematic biopsies are additionally recommended to maximize the diagnostic performance of the magnetic resonance imaging (MRI) diagnostic pathway for men with suspected prostate cancer (PCa) and positive scans. To reduce unnecessary systematic biopsies (SBx), MRI-directed approaches comprising targeted plus regional biopsy (TBx + RBx) are being investigated.

**Objective:**

To systematically evaluate the diagnostic performance of MRI-directed TBx + RBx approaches in comparison to MRI-directed TBx alone and TBx + SBx approaches.

**Evidence acquisition:**

The MEDLINE and Embase databases were searched according to the Preferred Reporting Items for Systematic Reviews and Meta-Analyses process. Identified reports were critically appraised according to the Quality Assessment of Diagnostic Accuracy Studies (QUADAS-2) criteria. Detection of grade group (GG) ≥2 PCa was the endpoint of interest. Fixed-effect meta-analyses were conducted to characterize summary effect sizes and quantify heterogeneity. Only MRI-positive men were included.

**Evidence synthesis:**

A total of eight studies were included for analysis. Among a cumulative total of 2603 men with suspected PCa, the GG ≥2 PCa detection rate did not significantly differ between MRI-directed TBx + RBx and TBx + SBx approaches (risk ratio [RR] 0.95, 95% confidence interval [CI] 0.90–1.01; *p* = 0.09). The TBx + RBx results were obtained using significantly fewer biopsy cores and avoiding contralateral SBx altogether. By contrast, there was significant difference in GG ≥2 PCa detection between MRI-directed TBx + RBx and TBx approaches (RR 1.18, 95% CI 1.10–1.25; *p* < 0.001).

**Conclusions:**

MRI-directed TBx + RBx approaches showed a nonsignificant difference in detection of GG ≥2 PCa compared to the recommended practice of MRI-directed TBx + SBx. However, owing to the extensive heterogeneity among the studies included, future prospective clinical studies are needed to further investigate, optimize, and standardize this promising biopsy approach.

**Patient summary:**

We reviewed the scientific literature on prostate biopsy approaches using magnetic resonance imaging (MRI)-directed targeted biopsy plus regional biopsy of the prostate. The studies we identified found arguments to potentially embrace such a combined biopsy approach for future diagnostics in prostate cancer.

## Take Home Message

It has been shown that limiting systematic biopsies to the proximity of magnetic resonance imaging (MRI)-positive prostate lesions is a promising diagnostic biopsy approach. However, considering the high degree of heterogeneity between the studies included in our review, prospective clinical trials are needed to further investigate and optimize this MRI-directed targeted plus regional biopsy approach.

## 1 Introduction

For biopsy-naïve men, the European Association of Urology (EAU) guidelines on prostate cancer (PCa) diagnosis currently recommend upfront magnetic resonance imaging (MRI) and then an MRI-directed targeted biopsy (TBx) plus systematic biopsy (SBx) approach in MRI-positive cases [1]. With this recommended biopsy approach, both the ipsilateral and contralateral lobes are still biopsied in a predominantly random systematic fashion, which is unique in the diagnostic process for solid-organ cancers [2], [3]. Most men benefit diagnostically from increased (perilesional/regional) sampling of the index lesion, while complementary SBx can increase unnecessary biopsy cores, potential harms, and patient burdens, and identify indolent cancers in men with false-positive MRI scans [4], [5], [6], [7], [8].

The concept of standard SBx is therefore widely disputed. MRI-directed biopsy approaches using TBx and only regional systematic biopsies (RBx) rather than standard SBx have been explored as an alternative approach to minimize biopsy cores, targeting errors, and grade migration. Use of such an MRI-directed TBx + RBx approach yields equivalent detection rates for significant PCa in comparison to the recommended MRI-directed TBx + SBx approach and reduces the number of biopsy cores and overdiagnosis rates [9], [10], [11], [12]. Currently, there is no consensus on such an MRI-directed TBx + RBx approach and several definitions can be found in the literature, including *focal saturation biopsy*, *perilesional biopsy*, *regional targeted biopsy*, and *targeted sector biopsy* [3], [11], [12], [13]. Although different interpretations exist, the scope of these interpretations is broadly in line: limit SBx to the proximity of the MRI-positive lesion. In this systematic review, we investigate the diagnostic performance of MRI-directed TBx + RBx approaches in comparison to MRI-directed TBx alone and TBx + SBx approaches.

## 2 Evidence acquisition

### 2.1 Objective

The aim of the review was to systematically evaluate the diagnostic performance of MRI-directed TBx + RBx approaches in detecting International Society of Urological Pathology (ISUP) grade group (GG) ≥2 PCa in comparison to MRI-directed TBx alone and TBx + SBx approaches in men suspected of having PCa and with positive MRI findings.

### 2.2 Study design

**Fig. 1:**
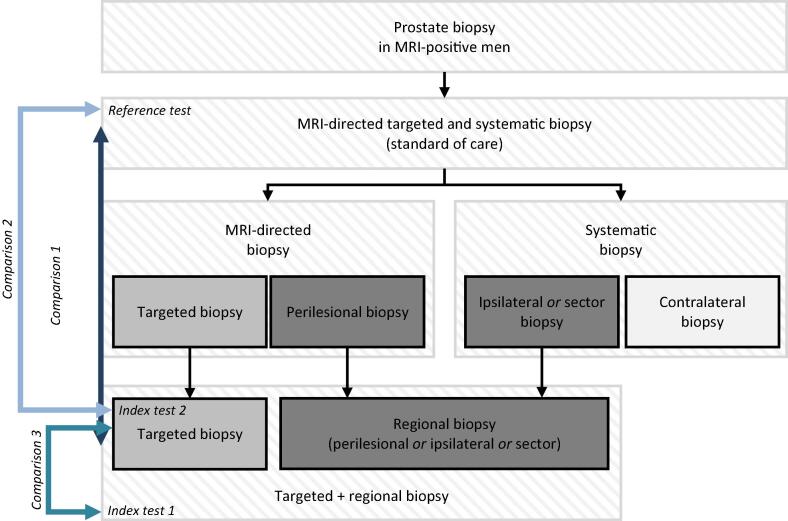
Definition of an MRI-directed targeted plus regional biopsy (TBx + RBx) approach: TBx with additional perilesional, ipsilateral, or sector biopsies. MRI = magnetic resonance imaging.

The study design in Fig. 1 shows the reference test (standard of care) using an MRI-directed TBx + SBx approach, index test 1 using an MRI-directed TBx + RBx approach, and index test 2 using an MRI-directed TBx approach. Given that there is currently no consensus on an MRI-directed TBx + RBx approach, different interpretations were included in this systematic review. RBx was defined as SBx in the proximity of the MRI-positive lesion, either via MRI-directed perilesional biopsy (biopsies around the index lesion), MRI-directed RBx (biopsies in the region of the index lesion), systematic ipsilateral biopsies (biopsies in the same lobe as the MRI-identified index lesion) or systematic sector biopsy (biopsies in the same sector as the MRI-identified index lesion).

### 2.3 Search strategy

A systematic search of Embase, MEDLINE (OvidSP), and Web of Science was conducted in March 2021 and updated in December 2021. The systematic literature search was performed with the help of an expert information specialist from the Medical Library of Antoni van Leeuwenhoek Hospital-Netherlands Cancer Institute, Amsterdam, The Netherlands. The free-text search terms used are provided in the Supplementary material. No limits were applied for searches. References from selected studies were also retrieved.

### 2.4 Inclusion criteria

The Population, Intervention, Control, and Outcomes (PICO) in this study were as follows. Patients suspected of having GG ≥2 PCa and undergoing prostate biopsies using a TBx + RBx approach were studied to evaluate the diagnostic performance in detecting GG ≥2 PCa in comparison to TBx alone and TBx + SBx approaches. To compare index and reference tests in the most objective manner, studies were only eligible if they reported individual biopsy results for all three biopsy approaches within the same patient. Studies were not limited to biopsy-naïve men. Studies that included men with a prior negative biopsy and men on active surveillance were also eligible.

We excluded studies that failed to evaluate the diagnostic performance of TBx + RBx approaches in comparison to TBx alone and TBx + SBx. We also excluded letters, editorials, study protocols, case reports, brief correspondence articles, and conference abstracts because comprehensive information is needed to correctly assess study quality and the study results.

### 2.5 Data extraction

**Fig. 2:**
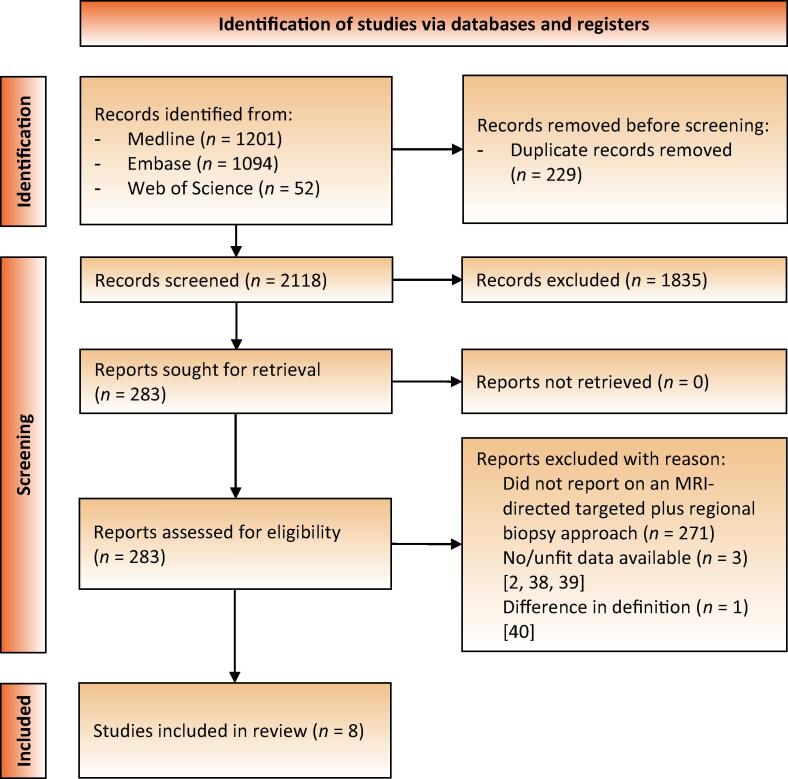
Preferred Reporting Items for Systematic Reviews and Meta-Analyses (PRISMA) flow diagram showing the search outcome and selection of full studies included in the review [3], [9], [10], [11], [12], [13], [18], [19] and the studies excluded [38], [39], [40].

We followed the Preferred Reporting Items for Systematic Reviews and Meta-Analyses (PRISMA) process for reporting the studies included and excluded, with the recommended flow chart showing the number of papers identified and included or excluded at each stage (Fig. 2) [14]. The abstract and full-text screening and subsequent data extraction were carried out by two reviewers (M.J.H. and M.F.S.) independently. Discrepancies between reviewers were resolved via discussion (M.J.H., M.F.S., and I.G.S.).

A data extraction form was developed to collect information on study methodology, patient characteristics, and MRI, biopsy, and pathology protocols. Positive MRI was defined as identification of a lesion suspicious for GG ≥2 PCa on the MRI scan with a score of 3–5 on a 5-point scale (Likert or Prostate Imaging-Reporting and Data System [PI-RADS]) [15], [16].

### 2.6 Assessment of study quality

The quality of the studies was reviewed according to the Quality Assessment of Diagnostic Accuracy Studies-2 (QUADAS-2) criteria [17].

### 2.7 Statistical analysis

The primary outcome was detection of GG ≥2 PCa. To synthesize the results, we performed a meta-analysis using RevMan version 5.3.3 (Cochrane Collaboration, London, UK). Three comparisons were analyzed (Fig. 1): comparison 1 was MRI-directed TBx + RBx versus MRI-directed TBx + SBx; comparison 2 was MRI-directed TBx + SBx versus MRI-directed TBx; and comparison 3 was MRI-directed TBx + RBx versus MRI-directed TBx. All meta-analyses were conducted using the random-effects model. Risk ratios (RRs) were calculated by dividing the GG ≥2 PCa detection rates for the index and reference tests. Heterogeneity was assessed using the I^2^ statistic. In addition, forest plots were constructed. Publication bias was assessed via visual inspection of funnel plots. To further synthesize the results, associations between different TBx + RBx approaches and the GG ≥2 PCa detection rate were investigated using univariate linear regression analyses. Analyses of total biopsy cores were performed using paired-sample *t* tests.

A value of *p* < 0.05 was considered to indicate statistical significance. All statistical analyses were performed using SPSS for MacOS version 27 (IBM, Armonk, NY, USA).

## 3 Evidence synthesis

Nine potentially eligible studies were identified by the first reviewer (M.J.H.) and seven potentially eligible studies were identified by the second reviewer (M.F.S.); an agreement rate of 78% was observed between the reviewers. Ultimately, eight studies were eligible for inclusion: three prospective cohort studies [9], [11], [18] and five retrospective cohort studies [3], [10], [12], [13], [19]. All the studies included investigated the performance of their proposed TBx + RBx approach relative to both TBx + SBx and TBx alone. Seven studies performed MRI/transrectal ultrasound (TRUS) fusion biopsies, whereas one study performed in-bore MRI-guided biopsies followed by systematic TRUS-guided biopsies (Supplementary Table 1). This systematic review comprises a cumulative of 2603 MRI-positive men, all suspected of having GG ≥2 PCa who underwent prostate biopsies using TBx + SBx approaches (Table 1). The overall GG ≥2 PCa detection rate was 46.1% (1199/2603; Table 2).

**Table 1:** Study characteristics: methodology, patient population, and imaging and biopsy protocols^a^

Study	Methodology		Patient population								MRI		Biopsy		
	Design	*n*	Age (yr)	Bx history	DRE^+^, *n* (%)	PSA (ng/ml)	PSAD (ng/ml/cm^3^)	Prostate volume (cm^3^)	MRI^+^ (*n*)	Lesion size (cm)	Protocol	MRI suspicion score	Navigation	TR/TP	Reference test
Barrett 2016 [3]	RS	76	68 (53–76)	BN: 13	NA	8.9 (0.8–53.2)	NA	43.2 (13.9–292.6)	76	1.09 (0.09–9.07)	3.0 T	PI-RADS 4: 33	MRI/TRUS fusion	TP	TBx + 24-core SBx
				PN: 37								PI-RADS 5: 56			
				AS: 26											
Bryk 2017 [9]	PS	211	61.0 (56–66)	BN: 124	44 (21)	5.3 (3.8–6.9)	NA	NA	134	NA	3.0 T + DCE	PI-RADS 2: 77	MRI/TRUS fusion	*NA*	TBx + 12-core SBx
				PN: 87								PI-RADS 3: 73			
												PI-RADS 4: 45			
												PI-RADS 5: 16			
Freifeld 2019 [10]	RS	116	63.7 ± 8.33	BN: 55	NA	10.36 ± 14.59	0.22 ± 0.29	54.12 ± 30.39	116	14.32 ± 9.50^b^	3.0 T + DCE	PI-RADS 3: 31	MRI/TRUS fusion	*NA*	TBx + 12-core SBx
				PN: 43								PI-RADS 4: 47			
				AS: 18								PI-RADS 5: 38			
van der Leest 2019 [11]	PS	317	65 (59–68)	BN: 317	176 (28)	6.4 (4.6–8.2)	0.11 (0.08–0.18)	55 (41–77)	317	NA	3.0 T + DCE	PI-RADS 3: 40	In-bore MRGB + TRUSGB	TR	TBx + 12-core SBx
												PI-RADS 4: 136			
												PI-RADS 5: 141			
Raman 2021 [12]	RS	971	64.5 ± 7.4	BN: 309	NA	8.4 ± 7.9	NA	60.8 ± 29.1	971	0.9 ± 2.2	3.0 T + DCE	PI-RADS 3: 415	MRI/TRUS fusion	TP	TBx + 12-core SBx
				PN: 659								PI-RADS 4: 380			
												PI-RADS 5: 176			
Park 2020 [13]	RS	212	65 (60–71)	BN: 97	NA	7 (5–10)	0.19 (0.12–0.27)	36 (28–50)	212	9 (6–13)^b^	NA	PI-RADS 3: 100	MRI/TRUS fusion	TP	TBx + 12-core SBx
				PN: 115								PI-RADS 4: 65			
												PI-RADS 5: 47			
Hansen 2020 [18]	PS	487	66 (60–69)	BN: 121	NA	7.2 (5.0–10.5)	0.14 (0.09–0.23)	46 (34–73)	487	0.50 (0.28–1.00)	1.5 T	Likert 3: 140	MRI/TRUS fusion	TP	TBx + 24-core SBx
				PN: 214							3.0 T	Likert 4: 164			
				AS: 152								Likert 5: 183			
Tschirdewahn 2021 [19]	RS	213	66 (61–71)	BN: 132	31 (15)	7.8 (5.6–10.3)	0.14 (0.09–0.21)	50 (40–65)	213	NA	3.0 T + DCE	PI-RADS 3: 210	MRI/TRUS fusion	TP	TBx + 24-core SBx
				PN: 81								PI-RADS 4: 168			
												PI-RADS 5: 54			

Bx = biopsy; MRI = magnetic resonance imaging; PS = prospective study; RS = retrospective study; DRE = digital rectal examination; PSA = prostate-specific antigen; PSAD = PSA density; TBx = targeted Bx, TRUS = transrectal ultrasound; TR/TP = transrectal/transperineal; BN = Bx-naïve; PN = prior negative Bx; AS = active surveillance; NA = not available; csPCa = clinically significant prostate cancer; PI-RADS = Prostate Imaging-Reporting and Data System; DCE = dynamic contrast enhancement; MRGB = MRI-guided biopsy; TRUSGB = TRUS-guided biopsy.

a All the studies used International Society of Urological Pathology grade group ≥2 as the definition for clinically significant prostate cancer. Data for continuous variables are reported as the median (interquartile range) or mean ± standard deviation.

b Lesion size in mm.

**Table 2:** Study outcome results: number of GG ≥2 cancers detected by the index tests

Study	Patients	Number of GG≥2 cancers detected, *n* (%)		
		Reference test	Index test 1	Index test 2
		(TBx + SBx)	(TBx + RBx)	(TBx)
Barrett 2016 [3]	76	67 (100)	60 (90)	52 (78)
Bryk 2017 [9]	211	49 (100)	47 (96)	36 (73)
Freifeld 2019 [10]	116	55 (100)	53 (96)	47 (85)
van der Leest (2019 [11]	317	180 (100)	179 (99)	159 (88)
Raman 2021 [12]	971	435 (100)	427 (98)	372 (86)
Park 2020 [13]	212	104 (100)	95 (91)	78 (75)
Hansen 2020 [18]	487	221 (100)	202 (91)	149 (67)
Tschirdewahn 2021 [19]	213	88 (100)	87 (99)	78 (89)
Overall	2603	1199 (100)	1150 (96)	971 (81)

GG = International Society of Urological Pathology grade group; TBx = targeted biopsy; SBx = systematic biopsy; RBx = regional biopsy.

### 3.1 Comparison 1: MRI-directed TBx+ RBx versus the reference test

**Fig. 3:**
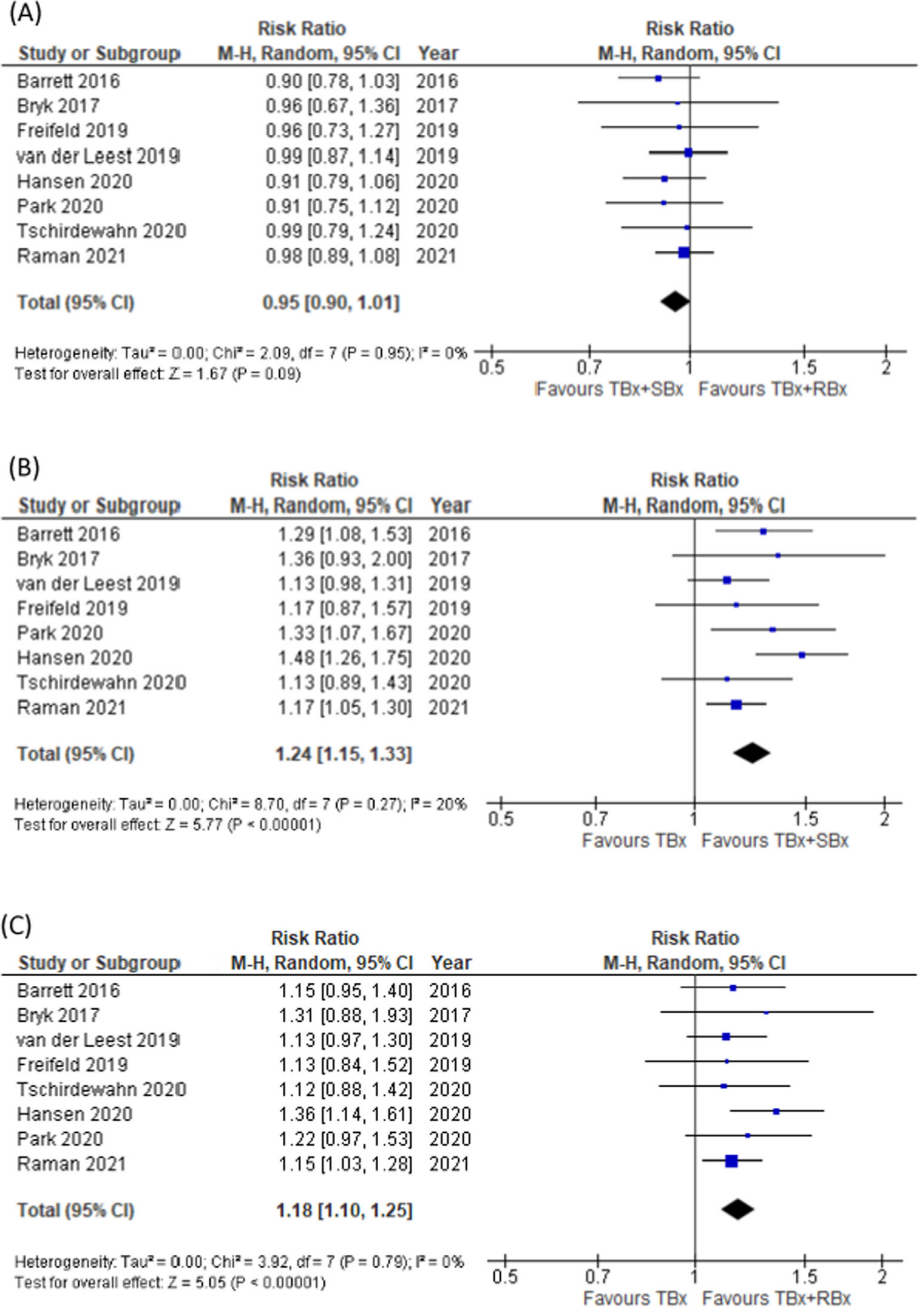
Forest plots for the detection rate of (A) targeted plus regional biopsies (TBx + RBx) in comparison to targeted plus systematic biopsies (TBx + SBx) and (B) TBx + SBx in comparison to targeted-only biopsy (TBx). (C) Forrest plot for the detection rate of TBx + RBx in comparison to TBx. CI = confidence interval; df = degrees of freedom; M-H = Mantel-Haenszel.

There was a nonsignificant difference in GG ≥2 PCa detection between MRI-directed TBx + RBx (index test 1) and the reference test of TBx + SBx (RR 0.95, 95%CI 0.90–1.01; *p* = 0.09; Fig. 3A). The number of cores was 9.5 (interquartile range [IQR] 7.5–12.3) for TBx + RBx and 16.5 (IQR 15.3–26.8) for TBx + SBx (mean difference 9.6, 95% CI 4.6–14.7; *p* = 0.003; Table 3).

**Table 3:** Study outcome results: number of biopsy cores

Study	Definition of index test 1		Total number of BCs (*n*)			BC
	RBx template	Test name	Reference test	Index test 1	Index test 2	Reduction^a^
Barrett 2016 [3]	4 target sector cores	Target sector biopsy	27	7	3	20
Bryk 2017 [9]	6 ipsilateral cores	Ipsilateral systematic biopsy	16	10	4	6
Freifeld 2019 [10]	6 ipsilateral cores	Ipsilateral systematic biopsy	15	8–9	2–3	6
van der Leest 2019 [11]	4 perilesional cores	Focal saturation biopsy	16	6–8	2–4	8
Raman 2021 [12]	Within a 2-cm penumbra	Regional targeted biopsy	17.0 ± 2.0^b^	13.2 ± 1.5^b^	5.0 ± 1.9^b^	4
Park 2020 [13]	2 adjacent sector cores	Focal saturation biopsy	15	4–5	2–3	10
Hansen 2020 [18]	Cores from adjacent sectors	Saturation targeted biopsy	26	10–20	2	6
Tschirdewahn 2021 [19]	Cores from adjacent sectors	Target saturation biopsy	28	9–10	4	18
Overall (IQR)	4–10 perilesional cores	Targeted plus regional biopsy	16.5 (15.3–26.8)	9.5 (7.5–12.3)	3.5 (3.0–4.0)	6.5 (6.0–16.0)

BC = biopsy core; reference test = targeted biopsy (TBx) + systematic biopsy (SBx); index test 1 = TBx + regional biopsy (RBx); index test 2 = TBx alone; IQR = interquartile range.

a BC reduction = (TBx + SBx) − (TBx + RBx).

b Mean ± standard deviation.

Detection of GG ≥2 PCa using MRI-directed TBx + RBx was visually best using a total of eight to 13 biopsy cores (Supplementary Fig. 1A). However, the total number of biopsy cores was not significantly associated with GG ≥2 PCa detection (*p* = 0.92; *B* = −0.03, 95% CI −0.86 to 0.79).

### 3.2 Comparison 2: MRI-directed TBx alone versus the reference test

There was a significant difference in GG ≥2 PCa detection between the MRI-directed TBx (index test 2) and TBx + SBx (reference test) approaches (RR 1.24, 95% CI 1.15–1.33; *p* < 0.001; Fig. 3B) in favor of the reference test. The number of cores was 16.5 (IQR 15.3–26.8) for TBx + SBx and 3.5 (IQR 3.0–4.0) for TBx (mean difference 16.5, 95% CI 11.3–21.7; *p* < 0.001; Supplementary Table 1).

Detection of GG ≥2 PCa using MRI-directed TBx was visually best using three to four biopsy cores (Supplementary Fig. 1B). However the number of biopsy cores was not significantly associated with GG ≥2 PCa detection (*p* = 0.07; *B* = 5.75, 95% CI −0.76 to 12.26).

### 3.3 Comparison 3: MRI-directed TBx + RBx versus MRI-directed TBx alone

There was a significant difference in GG ≥2 PCa detection between the MRI-directed TBx + RBx (index test 1) and TBx (index test 2) approaches (RR 1.18, 95% CI 1.10–1.25; *p* < 0.001; Fig. 3C) in favor of index test 1. The number of biopsy cores was 9.5 (IQR 7.5–12.3) for TBx + RBx and 3.5 (IQR 3.0–4.0) for TBx alone (mean difference 6.9, 95% CI 2.8–10.9; *p* = 0.005; Table 3).

### 3.4 Publication bias, study quality, and heterogeneity

Publication bias was assessed via funnel plot analysis (Supplementary Fig. 2). We found no strong evidence of publication bias on graphical inspection.

Most of the individual studies followed the Standards for Reporting of Diagnostic Accuracy (STARD) guidelines [20] and prostate Standards of Reporting for MRI-Targeted biopsy studies (START) guidelines [21]. We reviewed the reports according to the QUADAS-2 criteria [17]. The risk of bias was assessed in a qualitative manner. The overall quality of the individual diagnostic studies should not be considered as high. We concluded that the overall methodological quality of the studies was moderate. Supplementary Fig. 3 provides a detailed description and analysis.

We did not identify heterogeneity between study outcomes (Fig. 3). The recruitment of men with a suspicion of PCa was focused on positive MRI findings. These men all underwent biopsies. Patient characteristics (age, prostate-specific antigen levels, and prostate volume) were representative of the general PCa patient population.

### 3.5 Discussion

The current review synthesizes the evidence on MRI-directed TBx + RBx approaches for PCa diagnosis in MRI-positive men. Our analyses show noninferiority to the currently recommended MRI-directed TBx + SBx approach [1]. On the basis of these analyses, an MRI-directed TBx + RBx approach could potentially be a future alternative to current recommendations.

Although previous studies have shown that MRI-directed TBx is a promising biopsy strategy [22], [23], there are still substantial differences in GG ≥2 PCa detection when compared to saturation template biopsy [13], [24], [25], [26], [27]. Additionally found cancers are often in sextants adjacent to MRI-positive lesions, while systematic sampling of normal-appearing nonadjacent sectors does not alter risk stratification in the majority of cases. The main reasons for missing these systematically found GG ≥2 cancers are imprecise lesion registration (underestimation of true tumor volume) and targeting errors due to MRI and ultrasound/cognitive fusion inaccuracies [28], [29], [30], [31]. Additional SBx is therefore still advocated to improve GG ≥2 PCa detection, but at the expense of a higher overall number of biopsy cores [32], [33]. The proposed MRI-directed TBx + RBx approach may overcome these differences in GG ≥2 PCa detection, as imprecise lesion registration and targeting errors are covered by the RBx cores.

MRI-directed TBx + RBx substantially improves lesion detection, tumor characterization, and tumor volume estimation, and also reduces the total number of biopsy cores and false-positive MRI results, potentially reducing procedure time and pathologist workload. Owing to the inclusion of three studies in which a 24-core SBx template was used, the median number of biopsy cores was particularly high. The exact framing of an MRI-directed TBx + RBx approach still needs to be defined. The index lesion size, PI-RADS score, biopsy history, lesion location, and number of lesions can potentially influence this approach and should therefore be taken into account (ie, fewer biopsy cores may be considered for larger lesions and more biopsy cores for smaller lesions).

Reducing the number of biopsies by limiting SBx to the proximity of the MRI-positive lesion could potentially also reduce overdiagnosis rates. The studies included suggest a reduction of 5–19% in detection of GG 1 PCa, as RBx reduce the chance of finding indolent PCa in men with false-positive MRI scans [9], [18], [19]. At the same time, this reduction in biopsy cores did not significantly impact on histopathological concordance [12]. However, a substantial number of men are still at risk of grade migration; biopsy cores underestimate or overestimate the true Gleason score. As shown by Raman et al [12], use of a TBx + RBx approach increased the risk of grade inflation when compared to performing TBx or SBx alone. TBx and RBx may result in a grade shift [34], [35]. The 2019 ISUP consensus conference on PCa grading has overcome some of the issues raised, by aggregating Gleason scores for biopsy cores [36]. The strategy for histopathology analysis of perilesional biopsy cores needs to be further explored, as it touches not only on tumor characterization (sampling heterogeneity) and related grade shifts but also on targeting errors and volume estimations [37].

The major strength of this diagnostic meta-analysis is its focus on studies using biopsy sampling via MRI-directed TBx, RBx, and SBx in the same patient. However, this meta-analysis has several limitations that may reduce the strengths of the conclusions. Demonstrated differences in many of the variables in conducting MRI and biopsies may all contribute to study heterogeneity. Since there is no consensus on how to perform MRI-directed TBx + RBx, the definition of regional sampling differed greatly between the studies included. Both the number and location of biopsy cores were different in the studies, limiting the generalizability of the proposed approach, so the results need to be regarded with caution. Although it affects our efforts to synthesize the available evidence, heterogeneity does offer opportunities for increasing our knowledge and provides relevant additional information on the topic of interest. In addition, this retrospective study presents a diagnostic biopsy approach and focuses on reducing biopsy cores. On the basis of the studies included it remains unknown how an MRI-directed TBx + RBx approach impacts therapeutic choices. Whether it is preferable to decrease the number of biopsy cores in the diagnostic setting or improve prostatic mapping for treatment planning in cases in which prostate cancer is present still needs to be assessed. We did not register our review protocol before the article search process, and we therefore mention this as a limitation. Finally, eight studies were included in this meta-analysis and were predominantly retrospective in nature. Moderate quality on critical appraisal (QUADAS-2) limits the strengths of the conclusions that can be drawn.

## 4 Conclusions

This systematic review synthesized the evidence on the diagnostic performance of MRI-directed TBx + RBx approaches. In comparison to the current MRI-directed TBx + RBx approach recommended, a nonsignificant difference in detection of GG ≥2 PCa was observed, although the total number of biopsies was significantly reduced. Considering the high degree of heterogeneity between the studies included, the findings should be interpreted with caution. Future prospective clinical trials are needed to further investigate and optimize this MRI-directed TBx + RBx approach and to substantiate the argument to obviate standard SBx from the current recommended diagnostic work-up for MRI-positive men.

  ***Author contributions***: Marinus J. Hagens had full access to all the data in the study and takes responsibility for the integrity of the data and the accuracy of the data analysis.

*Study concept and design*: Hagens, Schoots, van der Poel, van Leeuwen.

*Acquisition of data*: Hagens, Fernandez Salamanca, Schoots.

*Analysis and interpretation of data*: Hagens, Schoots.

*Drafting of the manuscript*: Hagens.

*Critical revision of the manuscript for important intellectual content*: Schoots, Fernandez Salamanca, Padhani, van Leeuwen, van der Poel.

*Statistical analysis*: Hagens.

*Obtaining funding*: None.

*Administrative, technical, or material support*: None.

*Supervision*: Schoots, van der Poel, van Leeuwen.

*Other*: None.

  ***Financial disclosures:*** Marinus J. Hagens certifies that all conflicts of interest, including specific financial interests and relationships and affiliations relevant to the subject matter or materials discussed in the manuscript (eg, employment/affiliation, grants or funding, consultancies, honoraria, stock ownership or options, expert testimony, royalties, or patents filed, received, or pending), are the following: None.

  ***Funding/Support and role of the sponsor*:** None.
