# Supplementary material for: Diagnostic Performance of a Magnetic Resonance Imaging-directed Targeted plus Regional Biopsy Approach in Prostate Cancer Diagnosis: A Systematic Review and Meta-analysis
Source: Eur Urol Open Sci. 2022 May 2;40:95–103. doi: 10.1016/j.euros.2022.04.001 (PMC9079161; doi:10.1016/j.euros.2022.04.001)

**Supplementary material**

**Supplementary methods.** Search strategy by search engine used (Medline (Ovid), Embase and Web of Science)

**Medline (Ovid):**

((("Magnetic Resonance Imaging, Interventional"/ AND exp biopsy/) OR ("Image-Guided Biopsy"/ AND exp "Magnetic Resonance Imaging"/)) OR ((saturat* ADJ3 (focal OR target* OR focus*)) OR ((perilesional OR penumbra*) AND (biops* OR sampl*)) OR PLB OR STB).ti,ab,kf.)
AND
((exp "Prostatic Neoplasms"/) OR ((prostate OR prostatic) ADJ3 (cancer* OR carcinoma* OR adenocarcinoma* OR tumor* OR tumour* OR neoplasm*)).ti,ab,kf.)

**Embase.com:**
((('interventional magnetic resonance imaging'/exp AND 'prostate biopsy'/exp) OR ('prostate'/exp AND 'nuclear magnetic resonance imaging'/exp)) OR ((saturat* NEAR/3 (focal OR target* OR focus*)) OR ((perilesional OR penumbra*) AND (biops* OR sampl*)) OR PLB OR STB):ti,ab,kw)
AND
(('prostate tumor'/exp) OR ((prostate OR prostatic) NEAR/3 (cancer* OR carcinoma* OR adenocarcinoma* OR tumor* OR tumour* OR neoplasm*)):ti,ab,kw)

**Web of Science:**

TS=((saturat* NEAR/3 (focal OR target* OR focus*)) OR ((perilesional OR penumbra*) AND (biops* OR sampl*)) OR PLB OR STB)
AND
TS=((prostate OR prostatic) NEAR/3 (cancer* OR carcinoma* OR adenocarcinoma* OR tumor* OR tumour* OR neoplasm*))

**Supplementary Figure 1.** Proportion of detected GG≥2 PCa by the number of **a)** targeted-plus-regional biopsies (TBx+RBx), and **b)** targeted biopsies (TBx).


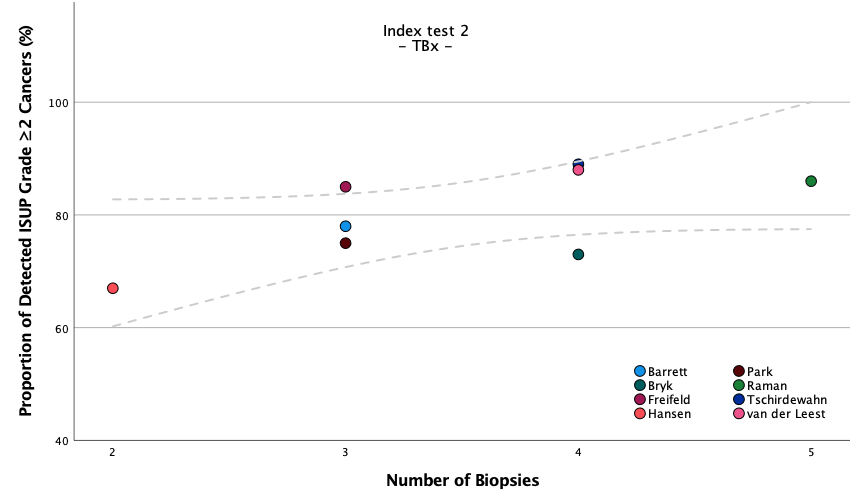

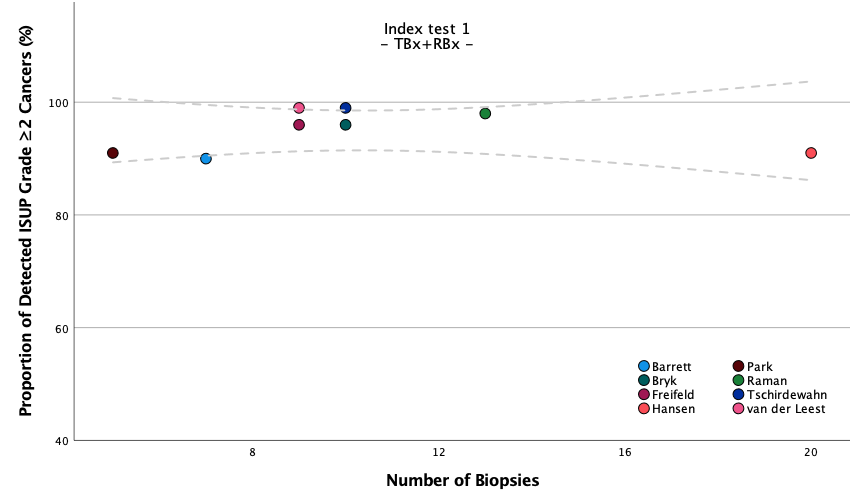


b

a

**Supplementary Table 1.** MRI/TRUS -fusion devices used by included studies

| Identifier | MRI/TRUS fusion devices |
| --- | --- |
| Reference |  |
| Barrett et al. (2016) [6] | Biopsee (Medcom) |
| Bryk et al. (2016) [12] | Artemis (Eigen) |
| Freifeld et al. (2019) [13] | UroStation (Koelis) / UroNav (Philips) |
| van der Leest et al. (2019) [14] | In-bore biopsy device (Invivo) |
| Raman et al. (2021) [15] | Artemis (Eigen) |
| Park et al. (2020) [16] | Artemis (Eigen) |
| Hansen et al. (2019) [21] | Biopsee (Medcom) |
| Tschirdewahn et al. (2020) [22] | MIM biopsy system (MIM Symphony Inc.) |

**Supplementary Figure 2.** Funnel plot analysis found no evidence for publication bias by graphical inspection.


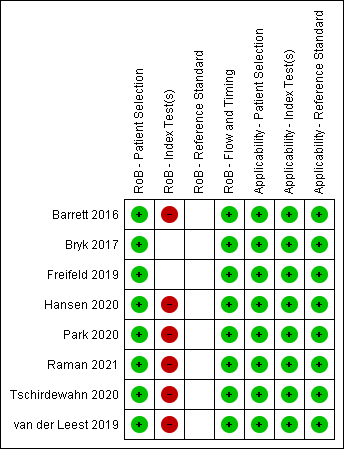

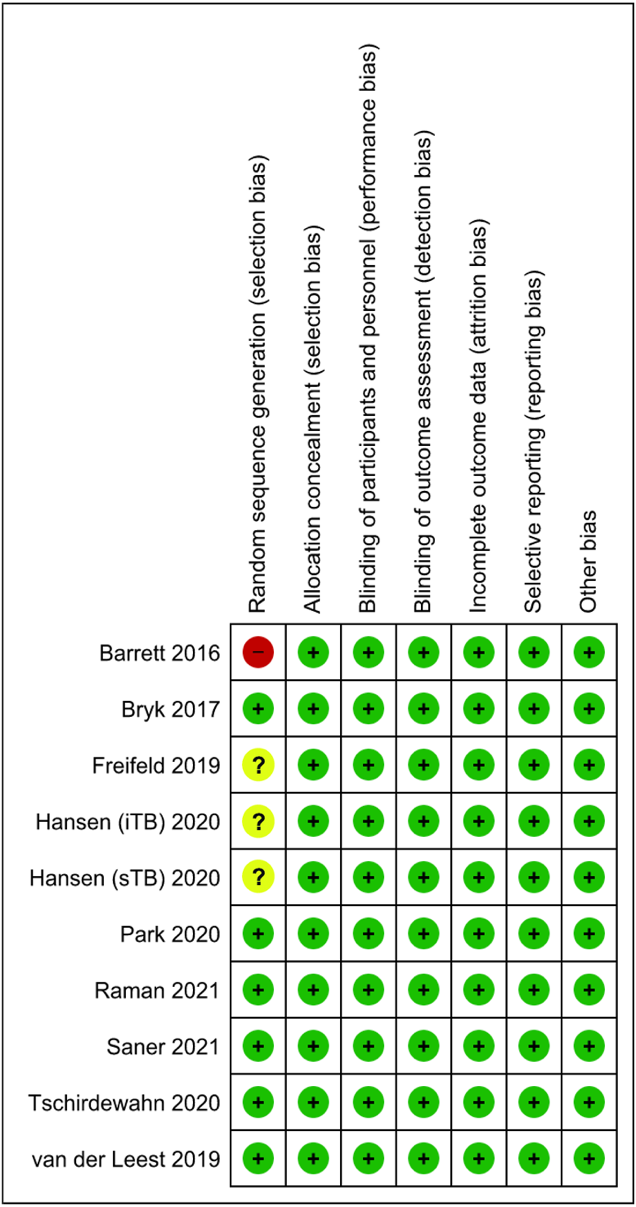

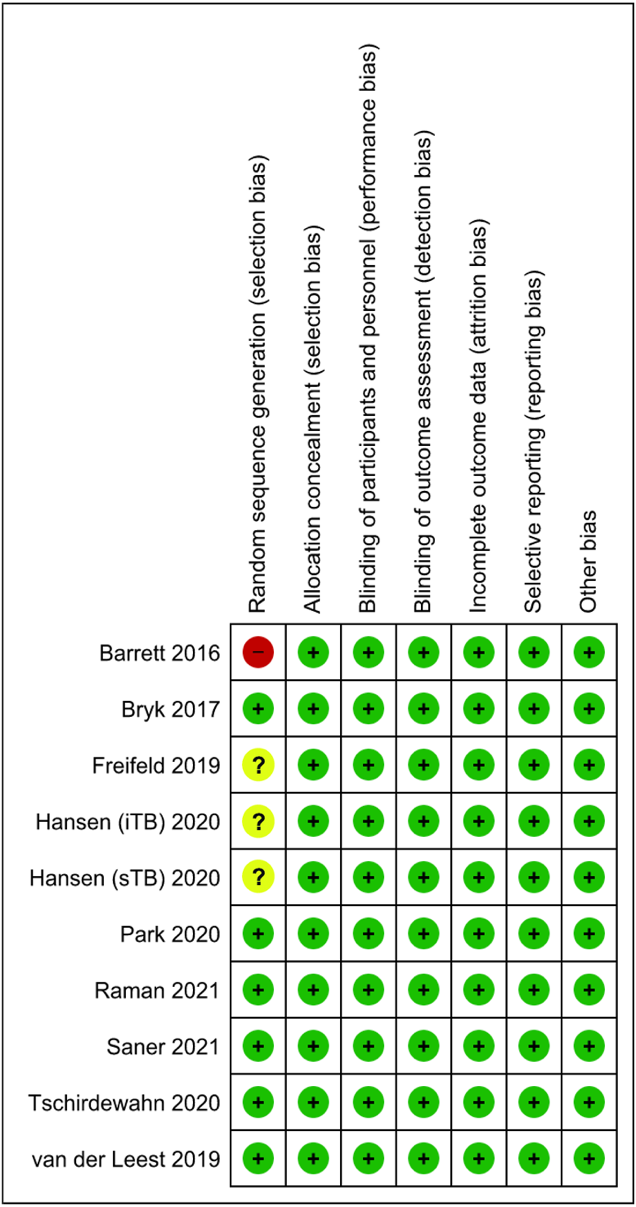

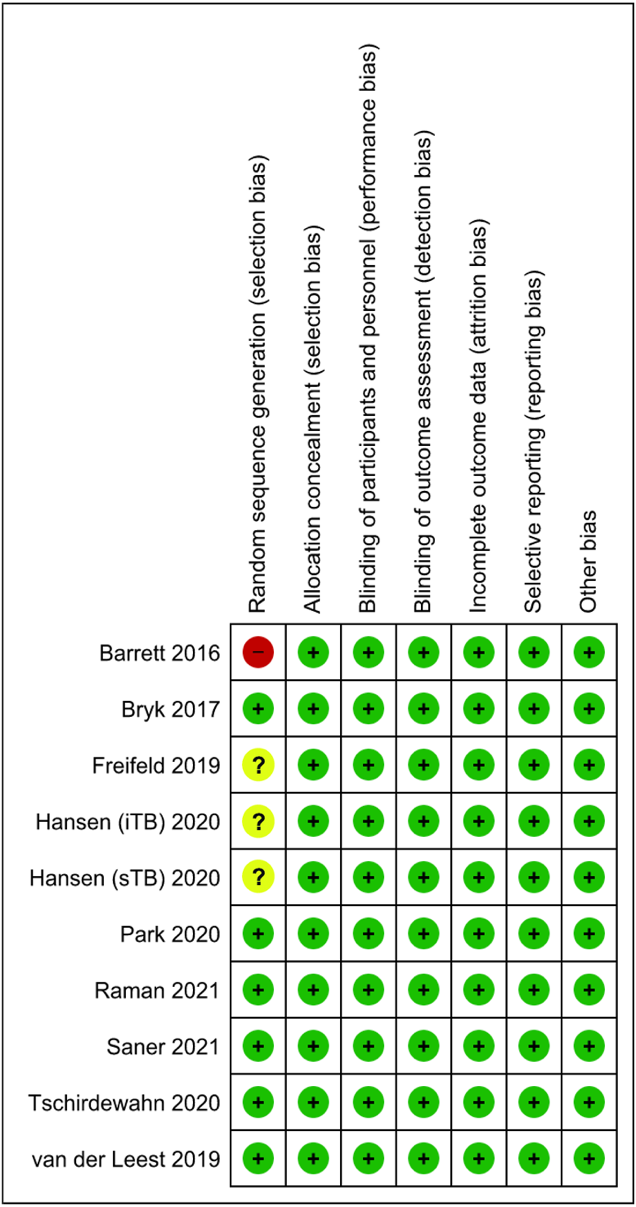

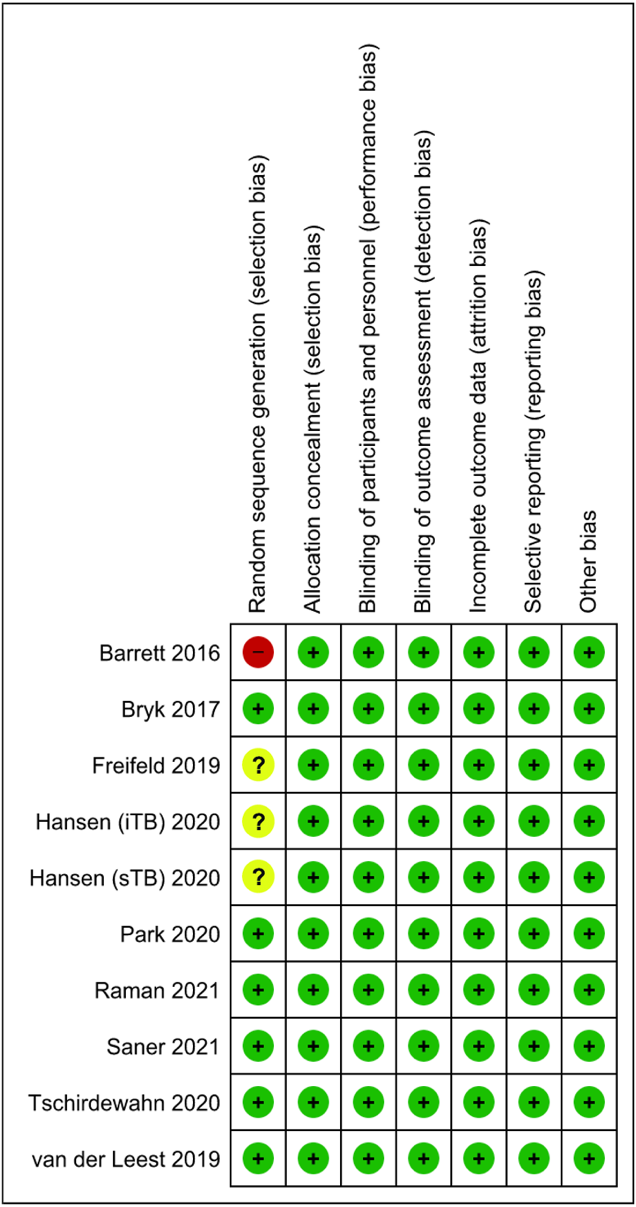

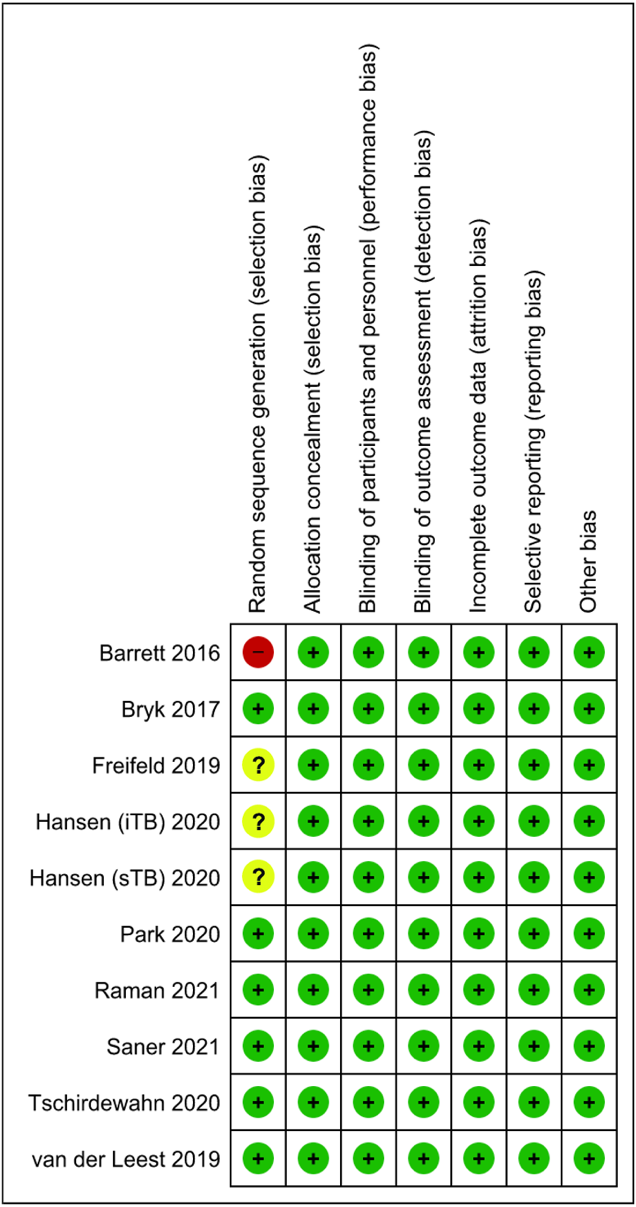

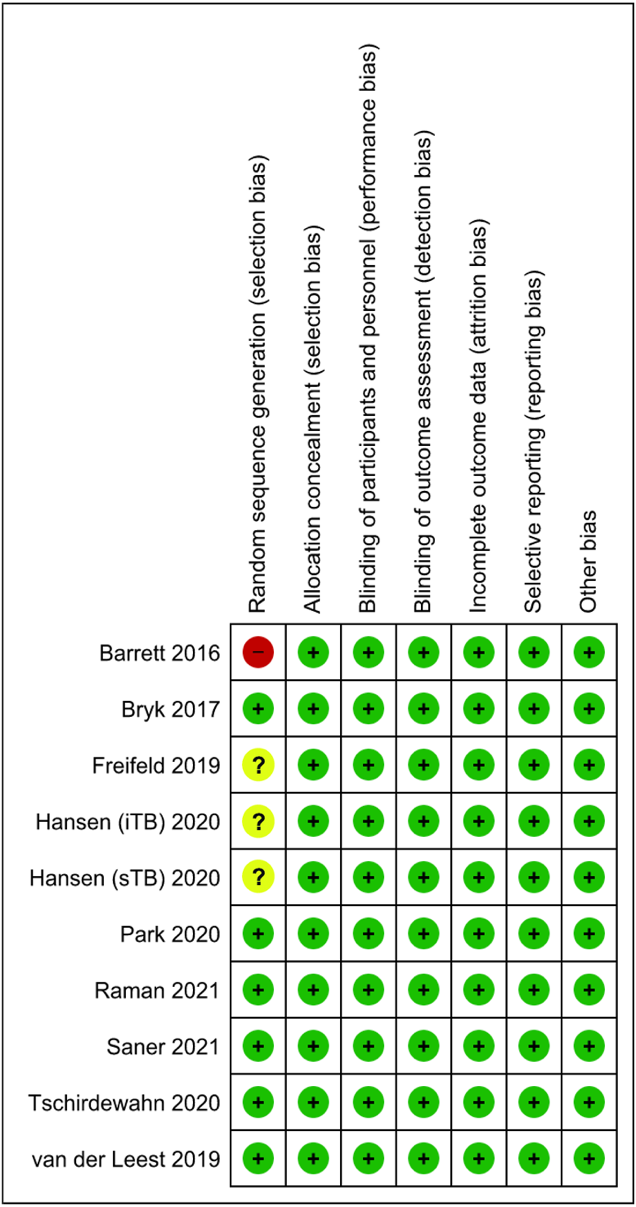

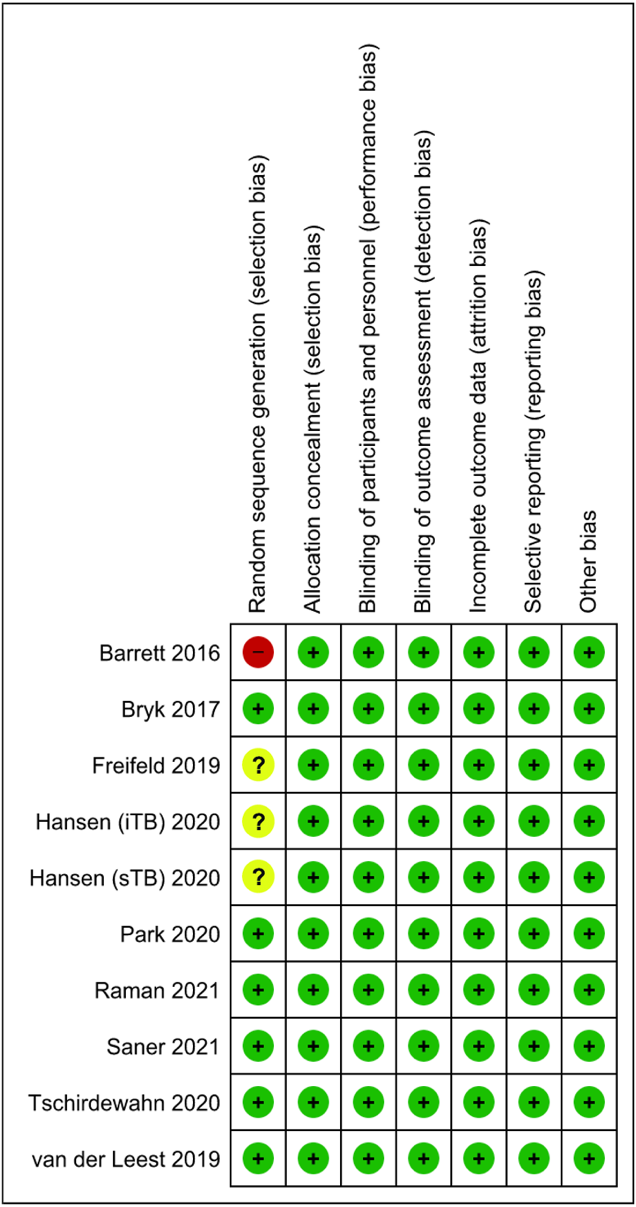

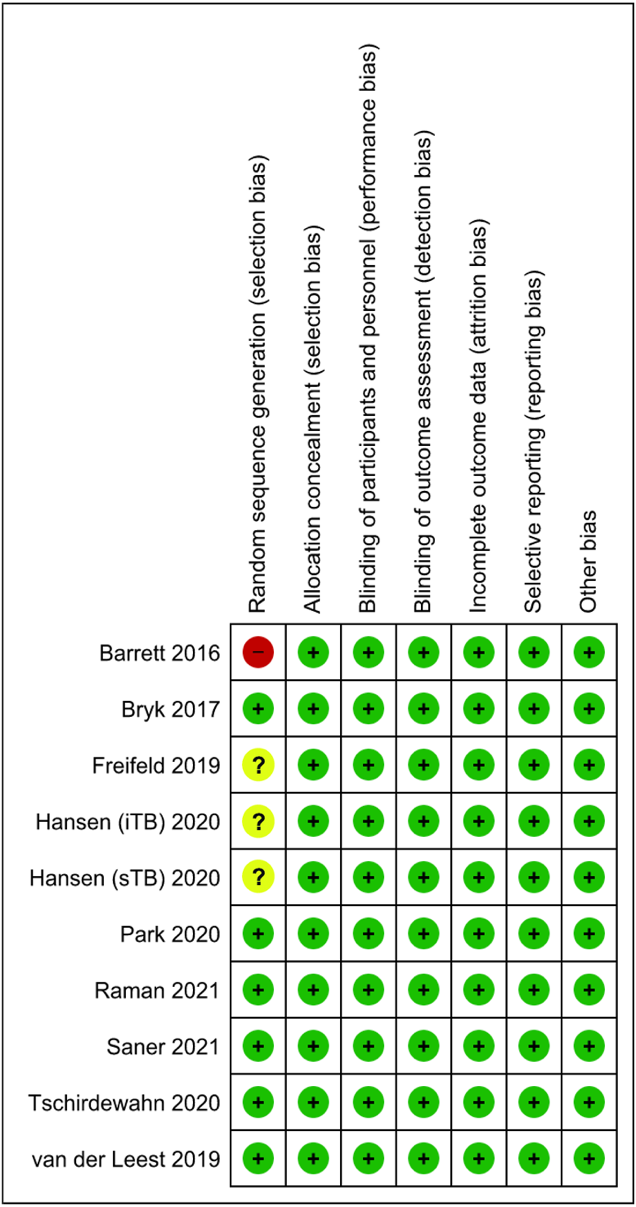

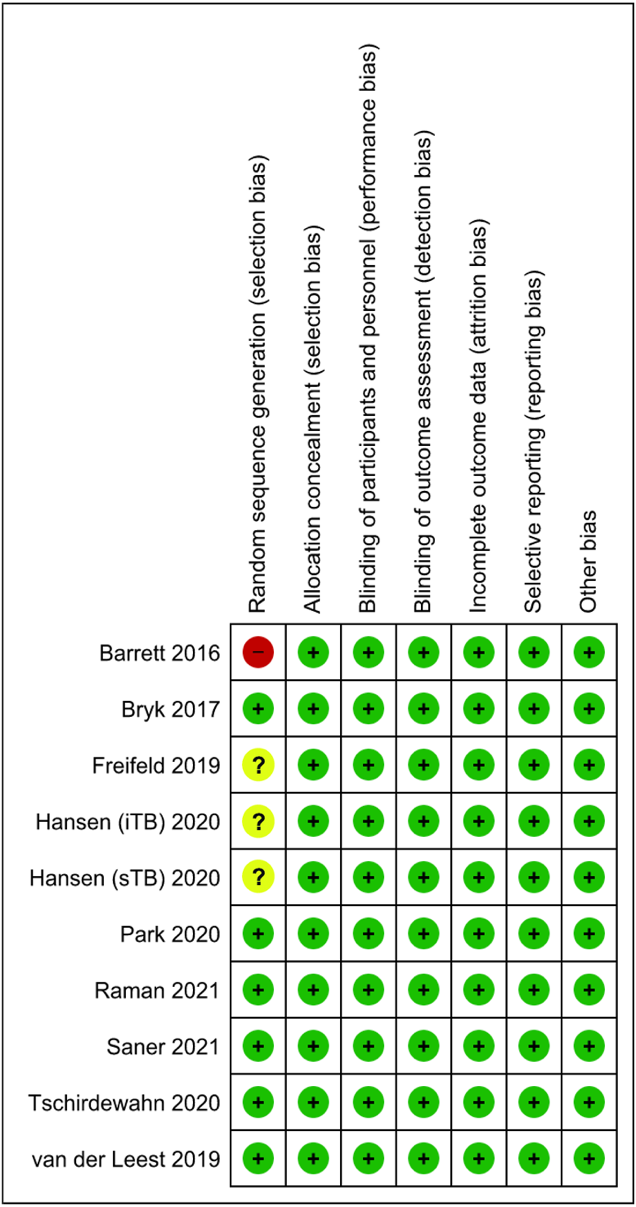

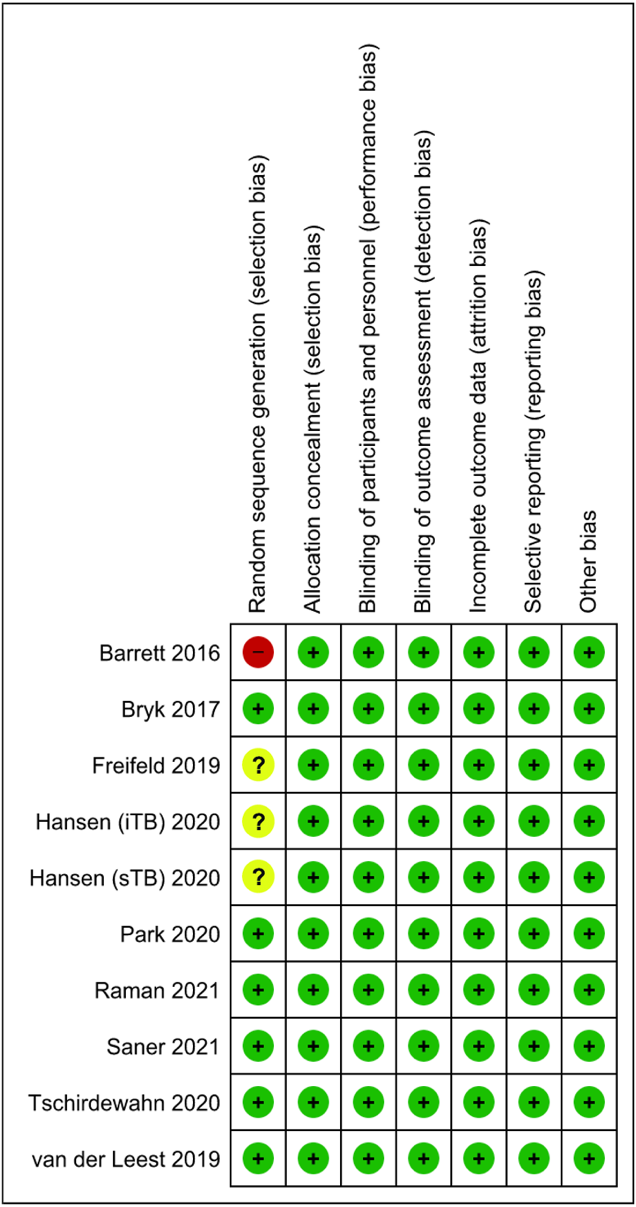

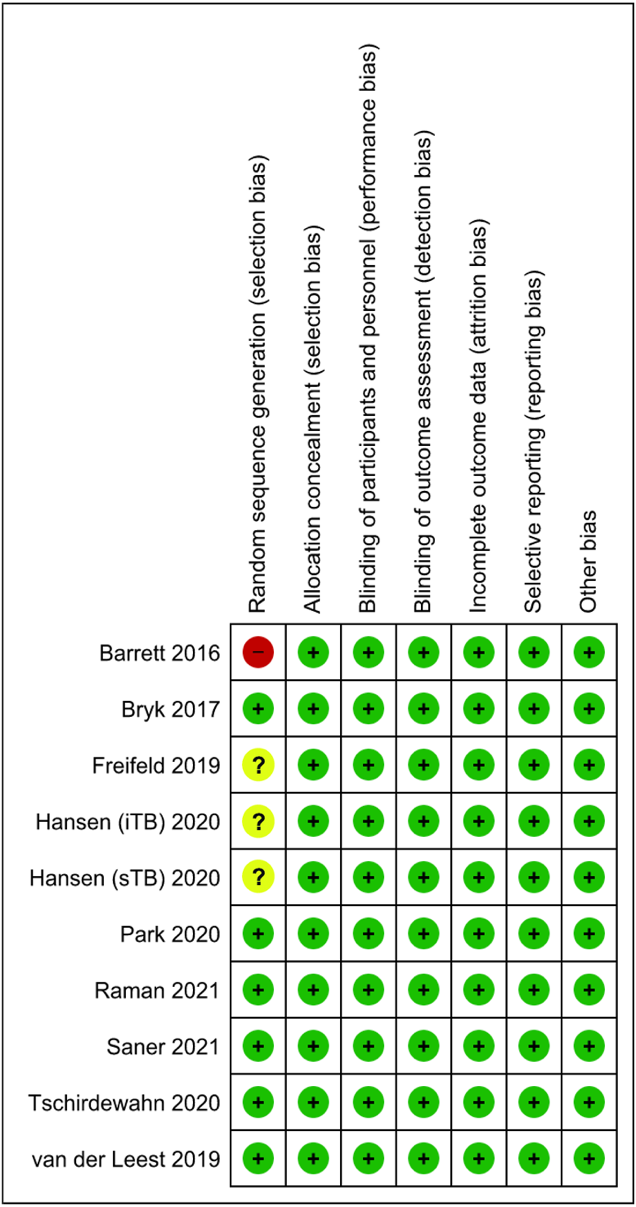

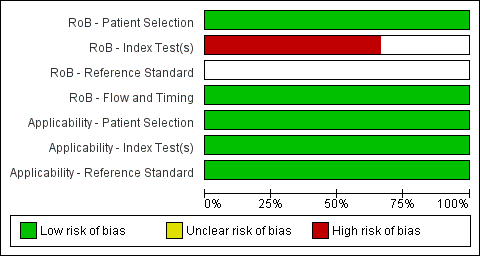


**Supplementary Figure 3.** Risk of bias summary and graph. Review authors’ judgment about each risk of bias item for each included study and presented as percentages across all included studies.


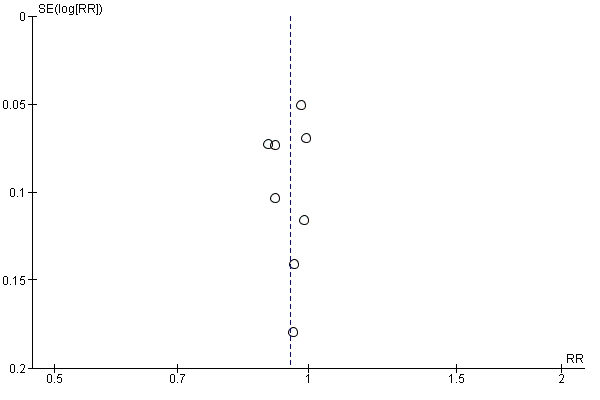

Supplement: Supplementary data 1 [file mmc1.docx]
